# Treatment status of extremely premature infants with gestational age < 28 weeks in a Chinese perinatal center from 2010 to 2019

**DOI:** 10.1007/s12519-021-00481-6

**Published:** 2021-11-12

**Authors:** Wen-Wen Zhang, Yong-Hui Yu, Xiao-Yu Dong, Simmy Reddy

**Affiliations:** 1 Department of Neonatology, Shandong Provincial Hospital Affiliated to Shandong First Medical University, Jinan, 250021 China; 2 Department of Neonatology, Shandong Maternal and Child Health Hospital, Jinan, 250021 China; 3 Cheeloo College of Medicine, Shandong University, Jinan, China

**Keywords:** Active treatment, Extremely premature, Infants, Mortality rate, Withdrawal of care

## Abstract

**Background:**

There is a paucity of studies conducted in China on the outcomes of all live-birth extremely premature infants (EPIs) and there is no unified recommendation on the active treatment of the minimum gestational age in the field of perinatal medicine in China. We aimed to investigate the current treatment situation of EPIs and to provide evidence for formulating reasonable treatment recommendations.

**Methods:**

We established a real-world ambispective cohort study of all live births in delivery rooms with gestational age (GA) between 24^+0^ and 27^+6^ weeks from 2010 to 2019.

**Results:**

Of the 1163 EPIs included in our study, 241 (20.7%) survived, while 849 (73.0%) died in the delivery room and 73 (6.3%) died in the neonatal intensive care unit. Among all included EPIs, 862 (74.1%) died from withholding or withdrawal of care. Regardless of stratification according to GA or birth weight, the proportion of total mortality attributable to withdrawal of care is high. For infants with the GA of 24 weeks, active treatment did not extend their survival time (*P* = 0.224). The survival time without severe morbidity of the active treatment was significantly longer than that of withdrawing care for infants older than 25 weeks (*P* < 0.001). Over time, the survival rate improved, and the withdrawal of care caused by socioeconomic factors and primary nonintervention were reduced significantly (*P* < 0.001).

**Conclusions:**

The mortality rate of EPIs is still high. Withdrawal of care is common for EPIs with smaller GA, especially in the delivery room. It is necessary to use a multi-center, large sample of real-world data to find the survival limit of active treatment based on our treatment capabilities.

## Introduction

Extremely premature infants (EPIs) with gestational age (GA) less than 28 weeks usually have a devastating prognosis; thus, the phenomenon of extremely premature birth is a major cause of neonatal death worldwide [1, 2]. In recent years, developed countries have conducted detailed discussions on whether to actively treat all live births in the delivery room (DR) [3–5]. China has also achieved some results in terms of active treatment, but the research subjects included only EPIs in the neonatal intensive care unit (NICU) [6–8]. A considerable number of infants who were withdrawn in the DR were not included. Furthermore, the impact of withdrawal of care on the mortality rate of all live EPIs in the DR is rarely reported [9]. This study collected data on the treatment of all live births in a provincial perinatal center with a large population base in China, which may provide a baseline for the treatment of EPIs.

The issue of withholding or withdrawal of EPIs medical treatment has been the subject of debate for decades, especially EPIs who are born near the limit of viability [10]. Nevertheless, there usually exists a framework and guidelines from the ethics committee of the professional societies outlining the principles of practice. Developed countries, such as France and the United Kingdom, have different recommendations of active treatment or comfort care for EPIs with GA between 22 and 24 weeks [5, 11]. Unfortunately, the situation may be completely different for resource poor countries, and especially for rapidly industrializing countries. Owing to differences in medical condition, economic foundation and cultural background, the minimum GA recommendation for active treatment in developing countries, such as China, is ambiguous. Presently, there is still a problem in that a considerable part of live-born EPIs with stable vital signs may not receive active treatment after delivery.

Our hospital is a provincial perinatal center in Shandong Province. Shandong Province’s fertility rate has consistently ranked first in China. With the establishment of a medical and health service system and a medical insurance system covering both urban and rural areas, the hospital is a provincial critical maternal care center and a provincial critical newborn care center. It plays the role of a provincial perinatal medical center. Because the definition of active treatment is not uniform, many EPIs who are worthy of treatment may not be treated actively.

The present study examined the treatment status of all live births with a GA of less than 28 weeks by establishing an ambispective cohort study in this perinatal center for 10 years (2010–2019). We distinguished whether the EPIs of all live births in the DR were actively treated, and we examined the treatment status of EPIs with a GA of less than 28 weeks based on real-world data.

## Methods

### Study design and population

This study obtained the information of infants born between 2010 and 2015 using the electronic medical record inquiry system and the death register retrospectively, while conducting a prospective cohort to collect medical information by EpiData entry software from 2016 to 2017 and then through the online database of Sino-Northern Neonatal Network (SNN) from 2018 to 2019. SNN is a clinical research database in China and has strict data-entry and quality-control standards. Trained data abstractors prospectively collected infant information at each NICU. Data were shared electronically each time to the Provincial Hospital affiliated to Shandong First Medical University. All live births of EPIs with GA between 24^+0^ and 27^+6^ weeks were collected from 2010 to 2019. Outcome assessments were completed at 40 weeks of corrected GA or before discharge. GA was assessed by early pregnancy ultrasound, obstetric examination, and obstetric history. If there was a 2-week difference between obstetric and pediatric assessments, pediatric assessments were used [12].

### Death category

Based on the place of death and the actual therapy infants received, we first divided the deaths into two groups: DR and NICU. Deaths in DR were divided further into (1) initial resuscitation failure (IRF) or (2) primary nonintervention (PNI) but simply receiving comfort care after birth [13]. Deaths in NICU were divided into: (3) redirection of care (ROC), including nonterminal ROC (when infants suffered severe neurological injury, which is defined as ≥ stage 3 intraventricular hemorrhage with ventricular dilatation according to the criteria of Papile et al. [14, 15]) and terminal ROC (when infants were classified as unstable due to presenting with any two of the following criteria: persistent desaturation despite 100% oxygen on mechanical ventilation, hypotension despite volume infusion and inotropes, protracted bradycardia or anuria for 24 hours or the score of clinical risk index for babies-II ≥ 15; necrotizing enterocolitis with multiple organ failure or bronchopulmonary dysplasia with respiratory failure was classified into this group [13, 16, 17]), the above-mentioned serious complications or terminal events occurred after active treatment; (4) socio-economic considerations (SEC): the infants’ guardians lacked the financial support or worried about possible severe sequelae, even when infants did not suffer severe neurological injury or in terminal status [18]; (5) maximal intensive care (MIC): life-sustaining therapies such as ventilatory and cardiovascular support and resuscitation efforts were pursued until death was pronounced [13, 16]. IRF, ROC and MIC were categorized as active treatment, while PNI and SEC were classified as withdrawal of care [19].

### End-of-life decisions

While in the DR or NICU, staff shared their professional medical opinion with parents in terms of the infant's condition and possible prognosis, after which the parents were allowed to make their final decision.

### Data collection

Data included GA, birth weight (BW), place of death, date of death, length of stay in either the DR or NICU, severe morbidity, diagnosis, outcomes, and reasons for withdrawal of care. All admission numbers of the children who met the inclusion criteria were also retrieved. The medical records were extracted from the electronic medical record system, and their admission records and mortality rates were reviewed.

### Statistical analysis

SPSS 22.0 statistical software package was used for data analysis. *χ*^2^ test or Mann–Whitney *U* tests were used to compare groups. The population attributable risk (PAR) was used to evaluate the impact of withdrawing care on the mortality rate. RR is relative risk. Pe represents the proportion of exposed persons in the population. PAR% = pe (RR-1)/[pe (RR-1) + 1] × 100%. Kaplan–Meier methods were used to build survival curves for different GAs, and survival rates were compared using the log-rank test. A two-sided *P* < 0.05 was considered statistically significant. Confidence intervals (CIs) were set at 95%. Statistical analyses were performed using GraphPad Prism version 8.0 software and SPSS statistical software version 22.0.

## Results

**Fig. 1:**
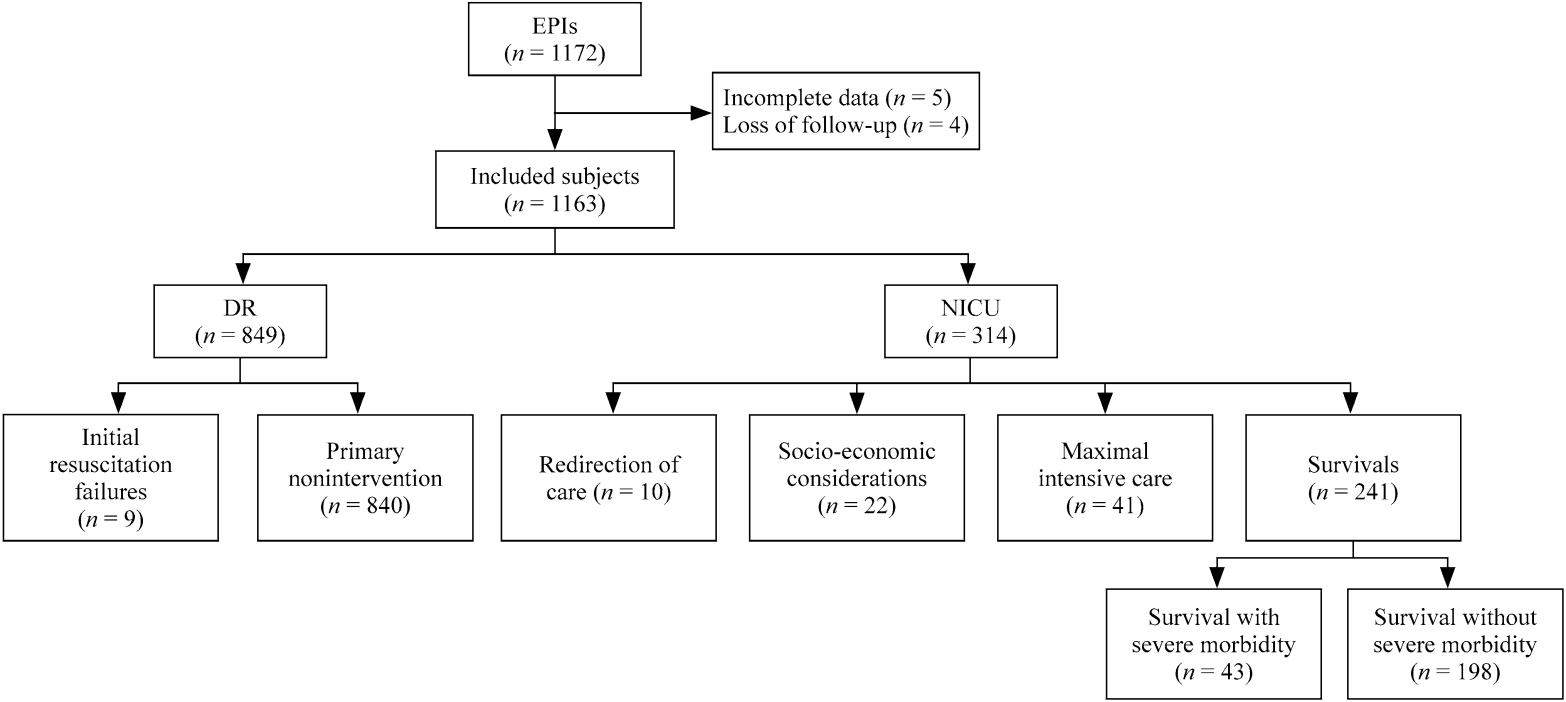
Flowchart of study population. *EPIs* extremely premature infants, *DR* delivery room, *NICU* neonatal intensive care unit

There were 1172 live EPI births during the study, excluding 5 with incomplete data, and 4 that were lost to lack of follow-up; thus, a total of 1163 infants were included for analysis, including 651 (56.0%) males, 512 (44.0%) females and 1 (0.9‰) hermaphrodite. Of the 1163 EPIs included in our study, 241 (20.7%) survived while 922 (79.3%) died. Among all survivors, 198 (82.2%, 198/241) survived without severe morbidity. There were 849 (73.0%, 849/1163) deaths in the DR, including 9 (1.1%, 9/849) cases of IRF and 840 (98.9%, 840/849) deaths due to PNI. There were 73 (6.3%, 73/1163) deaths in the NICU, among which 10 (13.7%, 10/73) died from ROC, 22 (30.1%, 22/73) died from SEC, and 41 (56.2%, 41/73) died from MIC. A flow diagram of infants included in the study is provided in Fig. 1.

All the included EPIs were divided into two groups and were stratified according to GA and BW. Compared with the active treatment group, infants in the withdrawal of care group in the DR and NICU had lower GA and BW (*P* < 0.001). There was also no significant gender-related difference between the two groups (*P* > 0.05) (Table 1).

**Table.1:** Comparing basic characters of extremely premature infants between active treatment and care withdrawal (*n* = 1163)

Variables	Care withdrawal (*n* = 862)	Active treatment (*n* = 301)	*Z* or *χ*^2^	*P*
GA (wk), M (P25-P75)	26 (25, 26.7)	27 (26.4, 27.4)	− 13.153	< 0.001
24^+0^–24^+6^, *n* (%)	206 (23.9)	9 (3.0)		
25^+0^–25^+6^, *n* (%)	246 (28.5)	25 (8.3)		
26^+0^–26^+6^, *n* (%)	221 (25.6)	121 (40.2)		
27^+0^–27^+6^, *n* (%)	189 (21.9)	146 (48.5)		
BW (g), M (P25-P75)	800 (670, 930)	900 (790, 1060)	− 8.267	< 0.001
500–599, *n* (%)	111 (12.9)	14 (4.7)		
600–699, *n* (%)	147 (17.1)	23 (7.6)		
700–799, *n* (%)	163 (18.9)	40 (13.3)		
800–899, *n* (%)	179 (20.8)	60 (19.9)		
900–999, *n* (%)	128 (14.8)	66 (21.9)		
≥ 1000, *n* (%)	134 (15.5)	98 (32.6)		
Male, *n* (%)	471 (54.6)	180 (59.8)	2.411	0.121

*GA* gestational age, *BW* birth weight

By calculating the mortality rate of different GA and BW as affected by withdrawal of care, we get the relative risk and the attributable risk percentage. Table 2 shows that the risk of death within the withdrawal of care group is higher than that in the active treatment group, and withdrawal of care is an important reason for death. In addition, calculation of attributable risk percentage indicates that regardless of GA or BW stratification, the proportion of total mortality attributable to withdrawal of care is high.

**Table.2:** Population attributable risk percentage of withdrawing care

Variables	Death rate (%)		RR (95% CI)	Pe	PAR%
	Care withdrawal, 862/862 (100)	Active treatment, 60/301 (19.9)			
GA (wk)					
24^+0^–24^+6^	206/206 (100)	3/9 (33.3)	3.000 (1.191, 7.558)	0.986	66.4
25^+0^–25^+6^	246/246 (100)	4/25 (16.0)	6.250 (2.546, 15.344)	0.984	83.8
26^+0^–26^+6^	221/221 (100)	25/121 (20.7)	4.840 (3.414, 6.863)	0.898	77.5
27^+0^–27^+6^	189/189 (100)	28/146 (19.2)	5.214 (3.737, 7.275)	0.871	78.6
BW (g)					
500–599	111/111 (100)	4/14 (28.5)	3.500 (1.529, 8.012)	0.965	70.7
600–699	147/147 (100)	6/23 (26.1)	3.833 (1.927, 7.627)	0.961	73.1
700–799	163/163 (100)	9/40 (22.5)	4.444 (2.501, 7.900)	0.948	76.6
800–899	179/179 (100)	16/60 (26.7)	3.750 (2.465, 5.705)	0.918	71.6
900–999	128/128 (100)	10/66 (15.2)	6.600 (3.729, 11.681)	0.928	83.9
≥ 1000	134/134 (100)	15/98 (15.3)	6.533 (4.101, 10.409)	0.899	83.3

*GA* gestational age, *BW* birth weight, *RR* relative risk, *CI* confidence interval, *Pe* proportion of exposed persons in the population, *PAR* population attributable risk. PAR% = pe (RR-1)/[pe (RR-1) + 1] × 100%

Table 3 shows the changes in survival rates and causes of death in two time periods. As time goes by, the survival rate improved and withdrawal of care caused by socioeconomic factors and PNI decreased significantly (*P* < 0.001). The number of EPIs receiving active treatment throughout the course also increased (*P* = 0.04), and there is no obvious difference in the remaining parts (*P* > 0.05).

**Table.3:** Changes in survival rates and causes of death in two time periods

Modes of outcome (*n* = 1163)	2010–2015 (*n* = 493)	2016–2019 (*n* = 670)	*χ*^2^	*P*
Survival (*n* = 241)	66 (13.4)	175 (26.1)	28.025	< 0.001
IRF (*n* = 9)	6 (1.2)	3 (0.4)	2.189	0.139
PNI (*n* = 840)	385 (78.1)	455 (67.9)	14.681	< 0.001
ROC (*n* = 10)	6 (1.2)	4 (0.6)	1.281	0.258
SEC (*n* = 22)	19 (3.9)	3 (0.4)	17.755	< 0.001
MIC (*n* = 41)	11 (2.2)	30 (4.5)	4.214	0.040

Values are *n* (%). *IRF* initial resuscitation failure, *PNI* primary nonintervention, *ROC* redirection of care, *SEC* socio-economic considerations, *MIC* maximal intensive care

**Fig. 2:**
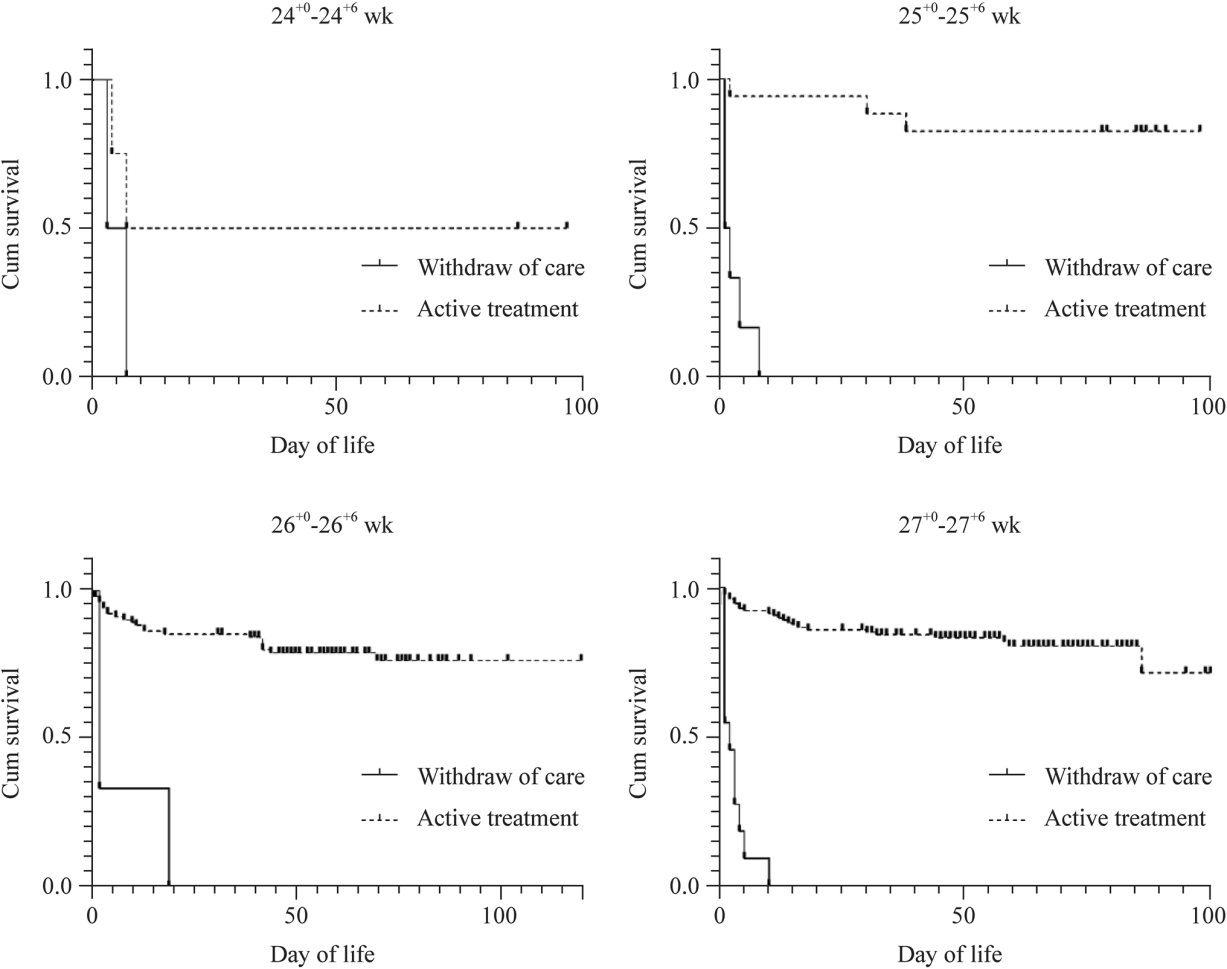
Kaplan–Meier survival estimates of different gestational age stratification

Figure 2 shows the survival curves of the four GA segments based on the age of death (in days). The log-rank test revealed no statistical difference in the hazard rate of death between the two groups of withdrawing care and actively treating EPIs with a GA of 24^+0^–24^+6^ weeks (*P* = 0.224, 95% CI 0.306–26.94). According to the results of the log-rank test, active treatment can significantly prolong the survival time of EPIs over 25 weeks (*P* < 0.001).

## Discussion

In China, there is no unified recommendation on the active treatment of the minimum GA in the field of perinatal medicine. In this ambispective cohort study, we examined the treatment status of EPIs with GA < 28 weeks in a Chinese perinatal center from 2010 to 2019.

With steady improvement of the treatment capability, many EPIs can be treated on time [20], but their early mortality is also relatively high [21]. Studies published by many developed countries’ neonatal collaboration networks showed that the mortality rates in EPIs ranged from 45.7 to 84% [3, 22–25]. The mortality in our study was 79.3%, of which 74.1% was withdrawn care. This suggests that withdrawal of care has an unavoidable impact on mortality of EPIs in our center. In recent years, developed countries have been paying more attention to the ways of withdrawing care in the NICU, such as withdrawal of respiratory and cardiac support [26]. However, in developed areas in Asia, such as Hong Kong of China, the most common mode of death in NICUs was active resuscitation [27]. In contrast, in the Chinese mainland, withdrawal of care is still chosen for many “alive” EPIs, which deserve more attention in the clinical field of perinatal medicine.

Our study showed that the proportion of withdrawing care was large and that most of the EPIs had a smaller GA and BW, especially in the DR. Through common sense, we reason that social economic cause may be the main factor contributing to the significant number of withdrawing care cases for EPIs in resource poor countries. Our study, however, includes astonishing data that indicate that more than 70% of the EPIs with GA < 28 weeks were not resuscitated in the DR for a variety of reasons. Perhaps this was more related to medical treatment recommendations. In France, the conventional policy is not to resuscitate before 24 weeks, so less than 1% of infants at 22 weeks to 23 weeks survive [14]. In Chinese obstetrics textbooks currently in use, preterm births are still defined as delivery with GA between 28 and 37 weeks [28], while in pediatric textbooks, preterm infants are defined as newborns with GA less than 37 weeks [29]. In contrast, many developed countries have treatment guidelines, and more than half of these guidelines state clearly that active treatment measures should be taken for EPIs with GA > 25 weeks [30]. A review in 47 highly developed countries also showed that a consensus was reached on the active treatment of EPI after 25 weeks [31]. Many countries also have established the treatment principle based on BW, which provides active treatment for infants with BW more than 500 g [32]. A British study showed that active treatment can improve the survival of EPIs significantly, and if perinatal medicine has a clear survival limit for active treatment, more newborns may have the chance to survive [33].

According to the Korean Neonatal Network, the survival rate of preterm infants of all GA and BW improved in 2015 compared to 2009 [34]. The survival rate for EPIs in Taiwan of China rose from 72% in 2007 to 78% in 2012 [35]. The Canadian Neonatal Network's average treatment success rate was 84.1% for EPI with GA between 24 weeks and 28 weeks in the past 10 years [36]. The survival proportion of the multi-center collaboration group in Guangdong province of China increased by 10.2% in 2013 compared with 2008 to 2012 [19]. In our study, the success rate of treatment after excluding withdraw of care reached 80.1% (241/301), and among all surviving EPIs, survival without serious complications accounted for 82.2% (198/241). Therefore, according to the development of the level of neonatal care, more and more EPIs younger than 28 weeks should be treated well. Each perinatal medical center should use real-word data to formulate the survival limit in their region to combine the treatment capabilities.

As for the reasons for withdrawing care, our preliminary investigation in previous studies showed that the main reason for parents' decision to withdrawal of care was not economic factors, but lack of communication or involvement of neonatologists in the decision-making prior to the delivery, leading to guardians’ lacking confidence in the treatment and concerns about poor prognosis [8]. In the present study, active treatment can prolong the survival time of infants over 25 weeks significantly. It is urgent to strengthen the communication between doctors and patients while promoting mutual understanding in the departments of obstetrics and pediatrics, so as to reduce withdrawal of care due to "worries about poor prognosis".

During the study period of 10 years, much has changed in the management of EPIs. Our hospital has updated the original concepts and techniques of respiratory support according to the 2014 American Academy of Pediatrics Guidelines for *Respiratory support at birth* and the 2016 European Guidelines for the *Prevention and Treatment of Neonatal RDS* [37, 38]. The optimized respiratory management strategy mainly includes the early application of non-invasive positive pressure breathing support within 30 minutes after birth, synchronized nasal intermittent positive pressure ventilation becoming the preferred non-invasive breathing mode, and high-frequency oscillating ventilation invasive breathing being widely used. Active use of glucocorticoids before childbirth to promote the maturity of the fetal lungs of premature infants [39]. Additionally, the feeding practices were modified according to the Canadian *Very low birth weight Infant Feeding Guidelines* [40]. At the same time, we took measures to keep warm to reduce the incidence of hypothermia when admitted to the hospital. In addition, we gradually strengthened communication with the obstetrics department and continued to build up the confidence of medical staff and parents in the treatment of infants. The implementation of these measures has provided a guarantee for the comprehensive treatment of premature infants and has improved the infant survival rate to a certain extent.

The strengths of our study are as follows: (1) this is the first time for a developing country, such as China, to demonstrate the death modes and the status quo of treatment for all live-born EPIs in the DR; (2) the 10-year data come from a provincial perinatal medical center with a high fertility rate, which is highly representative of the population in China. There are some limitations to our study: (1) this study uses a small sample of data from a single center to provide data and directions for the improvement of future treatment; (2) with a long span of 10 years, our treatment conditions and capabilities have undergone major changes, but the treatment situation has improved, which is encouraging; (3) from 2010 to 2015, it was a retrospective cohort study and failed to conduct a prospective investigation of the reasons for withdrawing care. We plan to collect multi-center data prospectively to find out the specific reasons for withdrawing care.

In conclusion, based on the data from our center, withholding or withdrawal of care has a great impact on the mortality rate of EPIs with GA less than 28 weeks. Therefore, it is necessary to formulate specific guidelines for the care of EPIs in China to avoid unnecessary withholding or withdrawing care in viable premature infants.

## Data Availability

The datasets generated during and/or analyzed during the current study are available from the corresponding author on reasonable request.
